# Next Generation Ingredients Based on Winemaking By-Products and an Approaching to Antiviral Properties

**DOI:** 10.3390/foods11111604

**Published:** 2022-05-29

**Authors:** Guillermo Pascual, María Dolores López, Marisol Vargas, Mario Aranda, Juan Antonio Cañumir

**Affiliations:** 1 Departamento de Producción Vegetal, Facultad de Agronomía, Universidad de Concepción, Vicente Méndez #595, Chillán 3780000, Chile; mlopezb@udec.cl (M.D.L.); marisolvargas@udec.cl (M.V.); 2 Laboratorio de Investigación en Fármacos y Alimentos, Departamento de Farmacia, Facultad de Química y de Farmacia, Pontificia Universidad Católica de Chile, Vicuña Mackenna 4860, Santiago 7810000, Chile; mario.aranda@uc.cl; 3 Laboratorio de Bioprocesos, Departamento de Agroindustría, Facultad de Ingenería Agrícola, Universidad de Concepción, Vicente Méndez #595, Chillán 3780000, Chile; jcanumir@udec.cl

**Keywords:** phenolic compounds, virus, wine waste, extraction techniques

## Abstract

Management of waste and use of winemaking by-products plays an important role in the development of new ingredients, especially with antiviral properties. Although the richness of bioactive compounds from wine waste is known, less is known about potential antiviral action. Bioactive compounds and health-enhancing effects of winery by-products make them potential candidates for use in antiviral ingredients. The design of new formulations by using nano-microencapsulation techniques will be necessary to successfully control diseases produced by viruses. Outcomes about the use of winery by-products, bioactive compounds found in winery wastes, green extraction techniques to concentrate these compounds, and development of formulations to obtain new ingredients were extracted from research around the world to be discussed and updated in this manuscript. The evidence collected in this review aims to encourage transfer of in vitro and in vivo knowledge to a new step for the development of antiviral and treatments.

## 1. Introduction

Wine production is one of the world’s oldest industries and one of the most important agricultural activities around the world, with an estimated surface area of 7.3 mha for the production of wine, table grapes, and raisins [1]. According to the International Wine Organization [2], 100 kg of grapes generate about 25 kg of waste, corresponding to skins (50%), stems, or rachis (25%) as well as seeds and liquids or semi-liquids (25%). In fact, the production of wine generates large amounts of solid and liquid waste by-products such as pomace, seeds, stems, waste from pruning, lees, and water, leading to a waste-management issue. The nature of the waste produced depends on the cultivar and specific vinification procedures used, which can also affect the properties of the residual material generated. Therefore, adequate waste treatment is needed to reduce the environmental impact of residues [3], and allow for a sustainable and environmentally friendly production.

**Figure 1:**
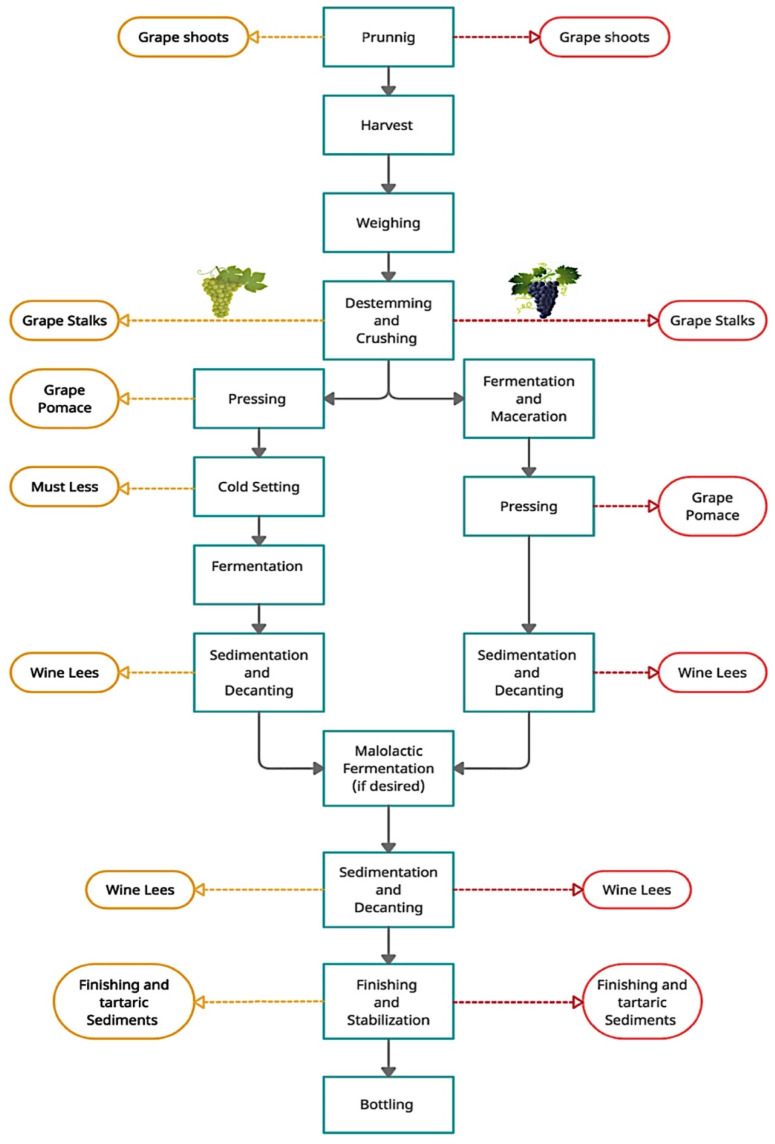
Schematic diagram of waste generation during wine production.

Figure 1 shows the different wastes produced during wine production.

Currently, a large part of the waste management in the wineries is aimed at composting pomace to be reintroduced into vineyards to maintain organic matter levels and comply with the increasing consumer demands for natural products and sustainable practices [4]. Most of the by-products generated during winemaking are rich in bioactive compounds and their impact on human health cannot be underestimated. This high content of bioactives opens a wide range of possibilities for different applications, from colorants for beverages and functional food, a source of biofuel, animal feed, and a special focus on the generation of natural medicines (Table 1).

Grape seeds mainly contain water (25–45%), glycidic compounds (34–36%), tannins (4–10%), nitrogenous compounds (4–6.5%), minerals (2–4%), lipids (13–20%), and lower concentrations of other substances such as sugars [5]. Total polyphenols in seeds can reach up to 60–70% of the extractable compounds, being a rich and natural source of antioxidants for the pharmaceutical, cosmetic, and food industries [6].

**Table 1:** Main by-products available in wine making and their uses.

By-Products	Bioactive Compounds	Current Use	Reference
Grape pomace waste	Organic matter content, polyphenols (anthocyanins and tannins), flavonol content, ethanol precipitate	Alternative source of antioxidant compounds and dietary fiber for yogurt	[7,8]
		Energy source	[9]
		To extend shelf life of lamb meat	[10]
		To reduce acrylamide formation	[11]
		To neutralize the production of reactive oxygen	[12]
		To reduce cholesterol level	[13]
		Stable delivery system, protecting resveratrol	[14]
		Biomethane	[15]
		Cosmetic formulation (skin aging)	[16]
		Dietary fiber supplement, human food supplement	[17]
Grape seed	Flavanol content. Lignocellulosic content	To modify the formulation of meat products	[18]
		Energy production, biodiesel	[19]
		Direct inclusion of natural antioxidants	[20]
		Skin moisturizer (gel formulation)	[21]
		Animal feed (rainbow trout)	[22]
		Extraction with supercritical CO_2_	[23,24]
Wastewater	Tartaric acid and malic acid content	Acidulant compound in soft drinks	[9]
Vine shoot and stems	Phenolic compounds	Biodegradable packaging	[25]
		Energy production, biomethane	[26]

The variety of applications detailed in Table 1 shows the applicability of the by-products of the wine industry, maximizing efficiency and generating economically viable alternatives such as the generation of biofuel, a problem that is becoming important with respect to a non-renewable resource such as fossil fuels.

On the other hand, grape stems, which can be partially or totally part of the fermentation and/or pressing process depending on the vinification procedure used, are a good source of proanthocyanidins [27], providing astringency to the resulting wine. The commercial value of stems is low, and they are usually recycled as organic fertilizers. There is also evidence that stalks are a rich source of bioactive compounds such as trans-resveratrol and derivatives, flavan-3-ols, and phenolic acid glycosides [28]. Vitis leaves are used in traditional medicine as laxatives, stomachics, diuretics, and refreshers, and also in palliative treatments of chronic bronchitis, heart disease, and gout [29].

Other wastes generated in the vineyard are after pruning (1 ton of biomass waste per hectare) [30], which are suitable for energy valorization, and mainly composed of cellulose and lignin with a low moisture content and high C/N ratio. Although biomass waste is an excellent source of bioenergy, its uses are still limited. In some areas, it is crushed and mixed with the soil as a fertilizer. Therefore, the roasting of these residues can be a profitable option to improve their fuel properties [31].

Finally, lees and winery wastewater are by-products that could be reused, although both present some inconveniences. The composition of wine lees is highly variable and depends on the winery process, whereas winery wastewater presents low pH, but is high in both sulphides and sodium content, and it also presents a high organic matter content.

Although the properties attributed to winemaking by-products as phenolic compounds have been extensively studied, specifically their antiviral capacity has not been developed significantly and there are limited antiviral products or ingredients made from wine industry residues. The scientific community has been concerned about the growing number of foodborne illnesses caused by some pathogens, which has led to the development of safe antimicrobial compounds derived from novel plants, including those present in grapes and grape products [32]. In this sense, winery waste by-products constitute a good source of natural polyphenols and antioxidants, which are considered completely safe compared with synthetic antioxidants. Therefore, this review summarized scientific information on the composition, richness, and functionality of mainly phenolic compounds present in waste by-products generated by the wine industry. It also focused on the development of some ingredients and advances on the preventive role that these compounds can play in the pathologies caused by viruses.

## 2. Updating of Bioactive Compounds Extracted from Winemaking By-Products

**Figure 2:**
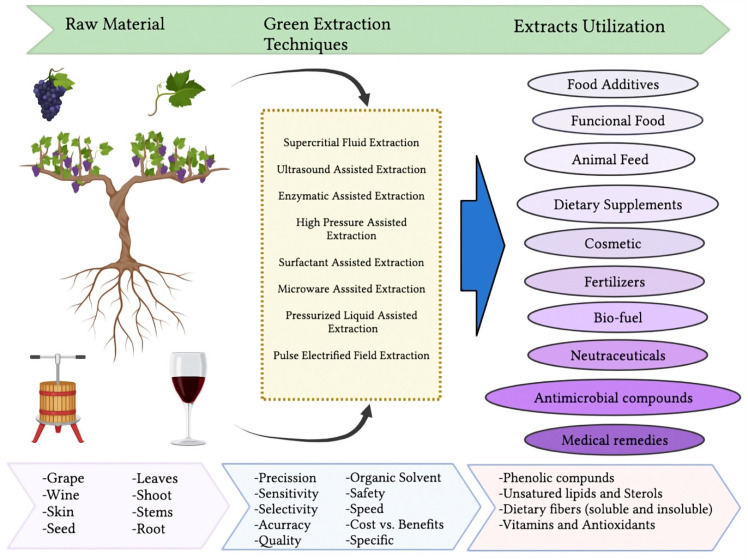
Green extraction techniques applied to by-products from the wine industry.

Most of the residues from winemaking are rich in phenolic compounds. Phenolic compounds in red wine pomaces comprise a diversity of chemical structures involved in the formation of the structure, color, transparency, and stability of the wine [7]. However, the composition of each pomace can vary depending on the grape variety of origin or growing conditions [8] and also the percentage of phenolic compounds that remains in the residues. Therefore, it is necessary to utilize an adequate extraction system where the losses are reduced, and it is more environmentally sustainable. The extraction of bioactive compounds presents some challenges because they differ in recovery yield and solubility. Recently, new extractive technologies have been applied to grape pomace in accordance with the principles of green chemistry such as supercritical fluid extraction (SFE), microwave-assisted extraction (MAE), microwave hydrodiffusion and gravity (MHG), ultrasound-assisted extraction (UAE), pulsed electric field (PEF), and ohmic heating (OH) since they are economic, innovative, and environmental-friendly processes [9] (Figure 2).

The use of conventional extraction technology such as solid–liquid extraction, heating, or grinding produces greater losses of bioactive compounds and environmental damage. Nevertheless, non-conventional technology such as pulsed electric fields, high voltage electrical discharges, pulsed ohmic heating, ultrasounds, microwave-assisted extractions, sub- and supercritical fluid extractions, or pressurized liquid extraction methods have already been applied for the extraction of high-added-value compounds from winery-processed samples [10].

Despite these techniques exemplifying a promising tool to recover high-added-value compounds from winery wastes and by-products, several considerations must be taken into account before choosing the technology, such as the matrix to be processed, the selectivity, the energy consumption, the equipment cost, and the value of the extract [10].

A study comparing the use of Pulsed Electric Fields (PEF) during alcoholic fermentation in the recovery of phenols compared with a thermal treatment for the Cabernet Franc variety showed a significant increase in the content of anthocyanins and tannins (approximately 51–62% compared with the thermal treatment) [11]. Additionally, the microwave-assisted extraction of grape pomace showed advantages as a remarkable decrease in extraction times, from 5 h to 5min, compared with a solid–liquid extraction method, increasing, as well, the content of acyl derivates not detected in the conventional method [10].

The use of the potential antioxidant capacity present in the by-products produced in the wine industry has been widely studied and information is available regarding the content of phytochemicals that promote human health. The phytochemicals present in the wine wastes are presented in Table A1.

Phenolic acids are the most prominent class of bioactive chemicals grouped under phenolic compounds present in various plant sources such as fruits, vegetables, spices, grains, and beverages [12,13]. These compounds frequently appear in a conjugated form, namely as glycosylated derivatives or esters of quinic acid, shikimic acid, and tartaric acid [13,14]. The genotype appears to be the major factor influencing the relative concentrations of the different phenolic compounds [33]. Flavonoids belonging to this group, such as flavan-3-ols, have a nuclear molecular structure of C6-C3-C6 and differ in the degree of oxidation of the central pyran ring [15]. They are abundantly found in seed and skin residues and play a very important role in the organoleptic properties of wines [34].

Anthocyanins, malvidin, petunidin, cyanidin, peonidinm and delphinidin (in the form of 3-O-glycosides) are the flavonoids responsible for the characteristic red color pigmentation. They are produced during ripening and are mainly found in grape skins. They are susceptible to light, temperature, oxygen, and pH [35]. Kaempferol, quercetin, myricetin, and isorhamnetin are the most abundant flavonols found in grapes, wine, and in the main by-products.

Hydroxybenzoic acids are derivatives of benzoic acid with a framework of seven carbon atoms of C6–C1 structure. Gallic acid can be found abundantly in grape stems, skins, and seeds, followed by syringic acid in grape stems, and protocatechic acid in grape seeds and skins [16].

Hydroxycinnamic acids are the derivatives of cinnamic acids having the framework of the C6–C3 structure. The most common hydroxycinnamic acid and its derivatives are p-coumaric acid, cinnamic acid, caffeic acid, ferulic acid, sinapic acids, isoferulic acid, and p-hydroxycinnamic [13]. The trans conformation of some phenolic acids (e.g., resveratrol) is naturally occurring, while the cis conformation is induced by UV exposure [36]. All these bioactive compounds must be protected and transformed into new ingredients; consequently, the use of encapsulation techniques is necessary.

Nevertheless, it is important to point out that the content of these molecules aforementioned vary depending on the starting material and the extraction technique used.

Therefore, current trends in extraction procedures from winery wastes and by-products will keep developing to replace conventional technologies with non-conventional ones since they present clear advantages including handling, reduction of the processing time, energy, the reduction of harmful and expensive solvents, and the increase of the extraction yields.

Among the many applications that have been described to wine by-products, development of antiviral ingredients has been hardly established. Hence, green extraction techniques mentioned above, where the content of the bioactive compound of interest is enhanced, with greater safety and innocuousness, open great possibilities of work on the line of constituents with antiviral potential.

## 3. Development of Ingredients of Products Based on Winemaking Products

Encapsulation is the technology used to safeguard sensitive materials by packaging materials in the form of micro- or nanoparticles. Encapsulation efficiency and stability of the capsules is closely related to the selection of the wall material [37]. Maltodextrin (MD) is the most commonly used encapsulating agent due to its high water solubility, low viscosity, and low sugar content [17]. There are also other encapsulating agents that offer a viable alternative such as gum arabic, skim milk powder, ascorbic acid, among others, which are generally used in mixture with MD. It is important to note that the success of the encapsulation will depend directly on the encapsulating agent or mixture of these, the working conditions of the equipment (air inlet temperature, pump power, feed flow, etc.), and the material to be encapsulated. Some examples are shown in Table 2.

The technology involved in encapsulation is effective in masking the unpleasant odor of the extracts and the product is rapidly soluble in water, releasing about 100% of the bioactive extracts within a few minutes. Therefore, the final products show improved technological characteristics suitable for the manufacture of functional foods and food supplements [18].

The current extraction techniques aforementioned show benefits like reduction of the extraction time, number of unit operations, energy consumption, environmental impacts, economical costs, quantity of solvent, and waste production, aiming to guarantee safe and quality extracts and/or products, and being more efficient to recover the phytochemicals present in wine by-products [19] as well as to enhance the formulation and microencapsulation techniques to develop an ingredient to be used in the industry (Table 2).

**Table 2:** Main technologies for the encapsulation of winemaking wastes.

Raw Material	Technology	Process Variable/Formulation	Encapsulation Agent	Main Result	References
Dry grape residue pressed	Microcapsulation. Buchi B-290 spray drying (Buchi Labortechnic AG, Switzerland).	Spray drying with the main chamber of 165 mm diameter, 600 mm cylindrical height, and 1.5 mm nozzle diameter at four air inlet temperatures (120, 140, 160, 180 °C). The pump power was kept at 40% to maintain feed flow rate as 12 mL min^−1^, and air flow rate as 35 m^3^ h^−1^. During drying processes, the temperature of the feed mixture was 25 °C	Maltodextrin and gum arabic as coating material. Two different core: coating material ratios (1:1 and 1:2), three different maltodextrin: gum arabic ratios (10:0, 8:2, and 6:4)	Encapsulation efficiency 98.8% and 99.1% for core: coating ratios of 1:1 and 1:2. Highest yield (64.9%) MD:GA ratio 10:0, at temperature 180 °C	[20]
Agiorgitiko (*Vitis vinifera*) grape pomace	Spray drying (Buchi, B-191, Buchi Laboratoriums-Technik, Flawil, Switzerland)	Ratio of wall-to-core material of 8.8, an inlet air temperature of 189 °C, a drying air flow rate of 65%	Maltodextrin:skim milk powder (50:50)	Optimum values of encapsulation efficiency (92.49%) and yield (37.28%)	[21]
Dry grape residue pressed	Spray drying process Buchi B-290 equipped with a 1.5 mm nozzle diameter and 600 mm × 165 mm main spray chamber	Peristaltic pump set to 40% power, 12 mL min^−1^ feed flow rate, and 35 m^3^ h^−1^ air flow rate. The temperature of the feed mixture kept constant at 25 °C during drying process.	Maltodextrin dextrose equivalents (MDDE4-7 and MDDE17-20) and gum Arabic (G9752)	The microcapsules obtained under optimal conditions were stored at two different relative humidities (33% and 52%) during 75 days.	[22]
Byproducts (seeds and peels) of Bordo red grapes (*V. labrusca*)	Pilot spray drying model MSD 5.0; freeze-drying in the proper equipment model LC 1500	Used a 2 mm nozzle and air flow of 40 L/min. The compressor air pressure was 0.2 MPa and the feed rate of the mixture 44 mL/min, performed by a peristaltic pump. Variables tested were inlet air temperature (130, 150, and 170 °C)	The carrier agent used in the atomization process was maltodextrin MOR-REX^®^ 1910	Bordo grape extracts using maltodextrin produced powders with low moisture content, low hygroscopicity, high solubility, and stable color.	[23]

The use of mixtures of encapsulating agents increases the efficiency and prolongs the durability of the encapsulation, as detailed in the works in the table above. One of the main advantages of encapsulation is to maintain and/or improve the shelf life and stability of bioactive compounds. In summary, encapsulation can generate a strategy to improve the stability of these compounds, and therefore the development of value-added foodstuffs to meet the growing consumer demand in the food, agricultural, and pharmaceutical industries [24].

One investigation carried out in grape pomace compared different types of maltodextrin and concluded that the choice of agent plays a crucial role in the storage stability of polyphenols, showing maltodextrin DE4-7 as significantly better in protection than maltodextrin DE17-20 in all conditions. Moreover, under identical experimental conditions, the stability of microencapsulated polyphenols was much higher at a relative humidity of 33% than at 52% [22].

The mixture of encapsulate material represents an alternative to improve the compound stability. Another study used protein concentrate (WPC), maltodextrin (MD), and gum arabic (GA) as encapsulating materials in differences preparation, WPC:MD/GA (5:0, 4:1, 3:2, and 0:5) followed by freeze drying. The grape seed extract [38] microcapsules coated with a WPC:MD/GA ratio of 4:1 and 3:2 with core-to-coat ratio of 1:5 were found to have the highest encapsulation efficiency (87.90–91.13%) and the smallest particle size with the maximum retention of antioxidant activity [39].

Hence, these are a source of functional compounds that can be exploited in the production of innovative foods and packaging, cosmetics, and also of new ingredients of the next-generation focus on viruses.

In fact, encapsulation of antivirals for food applications has been little explored. The lack of absorption of the bioactive compounds extracted from by-products of the wine industry limits their health benefits or their pharmaceutical use [25]. Their low stability is mainly attributed to poor absorption from the human gastrointestinal tract. Phenolic compounds found in wastes have to pass through the human gastrointestinal tract and be absorbed by enteric epithelial cells as they are administered orally. Furthermore, the extremely low pH (approximately 2.0) of gastric fluid and digestive enzyme can degrade these bioactive compounds in the human stomach [25]. Consequently, they have very low bioavailability. Hence, nanoparticle-based carriers present a great promise for control release since the passive transcellular pathway, the paracellular pathway, and endocytosis may be able to absorb nanoparticles loaded with bioactive compounds extracted from the wine industry.

Recent developments in the encapsulation of antiviral compounds include the use of chitosan to enhance the protection of epigallocatechin (-) gallate (a green tea polyphenol) [26]. This compound, microencapsulated, showed the potential to prolong antiviral activity against murine norovirus through gradual bioactive release combined with its protection against degradation under simulated physiological conditions. Therefore, these results highlight the potential of encapsulated natural antiviral compounds for use in food applications. Encapsulation of these antiviral compounds may provide enhanced and prolonged antiviral activity thanks to biopolymeric encapsulation matrices. Moreover, different studies demonstrated the efficacy of alginate-based release particles [40], suggesting that encapsulation could represent a viable tool for the transport and delivery of antiviral compounds.

## 4. New Insights against Disease and Viruses

Phenolic compounds from wine by-products play a protective role in plants that can be extrapolated to other living organisms. For humans, an excess of Reactive Oxygen Species (ROS) in the body can enhance the development of chronic non-communicable diseases such as cancer, cardiovascular disorders, neurodegenerative damage, Alzheimer’s disease, and inflammation in different organs [41]. Special attention is given to anthocyanins that contribute 90% of the antioxidant capacity of fruits, whereas the remaining 10% is attributed to flavonols, flavan-3-ols, and phenolic acids [42]. In addition, phenols exhibit chelating, anticancer, antimicrobial, and anti-inflammatory activities [43]. These capacities allow these phenols to react in biological systems, decreasing the occurrence of degenerative diseases associated with oxidative stress in tissues and organ systems [44]. This confirms that the continuous intake of food products with a high antioxidant content is associated with a lower incidence/severity of developing pathophysiological problems [41].

The target for valorization of these wastes is not only limited to remediating environmental problems, but also to utilize them as a source of functional ingredients. Valorization of waste from wineries provides commercialization of phenolic extracts, dietary fibers, and oil derived from grape pomace. Some of the residues and/or by-products generated in the production of wine have compounds with health-promoting properties, e.g., anthocyanins, which are highly concentrated in the pomace and have anti-inflammatory properties and antioxidant activity in human low-density lipoproteins [45], as well as positive effects on microcirculation diseases and ocular function [46].

Some studies have reported antiviral activity of phenolic compounds from grapes and grape products as well as from wine by-products (Table 3) [47].

Animal models are the main focus of studies to evaluate the in vivo antioxidant activity of phenolic matrices from winemaking wastes. Nowadays, there are biomarkers in urine and blood that are evaluated after the supply of the functional ingredient in rats, and even the intestinal microbiota, can be studied in relation to bioassimilation and/or bioavailability [57]. A study in rats showed that a high-cholesterol diet with 15% pomace incorporated halved liver and serum cholesterol levels, increased high-density lipoprotein by up to 26%, and had positive effects on microcirculatory disease and ocular function [58]. There is also evidence that grape seed extracts are a rich source of polyphenols, reducing the risk of heart disease by inhibiting Low Density Lipoprotein (LDL) oxidation, improving endothelial function, lowering blood pressure, preventing platelet aggregation, reducing inflammation, and activating proteins that prevent cellular senescence [59]. In addition, polyphenols from grape pomace increased the biodiversity degree of intestinal microbiota in broiler chicks [60], and improved the gain-to-feed ratio and overall performance in pigs [38,48]. Furthermore, grape pomace altered the nitrogen metabolism and decreased the ruminal ammonia production in male sheep [61] and modified the rumen microbial population involved in methane metabolism [62], enhancing the growth of facultative probiotic bacteria, and inhibited the growth of pathogenic ones in lambs [63].

Recent studies have shown that flavonoids exhibit antiviral activity against HIV, HSV, influenza virus (IV), RSV, severe acute respiratory syndrome coronavirus (SARS-CoV), measles, and rotavirus [64]. Resveratrol was also recently reported as inhibiting MERS-CoV infection by extending cell survival after virus infection and decreasing the expression of the nucleocapsid (N) protein, essential for MERS-CoV replication. This is supported by varied mechanisms of action, such as inhibition of adsorption, virus entry, virus binding, RTase, integrase, protease, inhibition of replication of DNA and RNA polymerases, and formation of protein complexes [65].

Grape extracts (skin and whole red grapes), grape juice, and wine were reported to inactivate various enteric viruses and herpes simplex virus (HSV) type 1 [49,66].

Bioactive compounds isolated from winemaking wastes, mainly flavonoids, may represent an interesting option against viral diseases. Indeed, flavonoids lack systemic toxicity, while their ability to synergize with conventional drugs has been widely demonstrated. Furthermore, they are considered as pleiotropic compounds, which indicates that multiple pathways where intercepted and also different cellular targets [67]. These characteristics make flavonoids potential candidates for interfering with the life cycle of coronaviruses [68]. To achieve maximum benefit from wine production waste extracts as an antiviral natural compound, more information is needed on the active compounds present in these extracts or isolated, the effect of the extraction method and the green extraction system used to obtain the bioactive compounds [49].

## 5. Future Perspectives

The worldwide demand for wine has been growing in recent decades, which has led to an increase in the use of winemaking by-products. The studies discussed in this review point out the potential of phenolic compounds present in wine and winemaking waste. Bioactivity, bioavailability, and toxicology of phytochemicals are based on the knowledge studied and provide a possibility of use as promoters of human health, dietary enhancers in animals, and other uses such as those exploited by the cosmetic industry. It is essential to perform tests in in vitro and in vivo conditions and to focus the work on the processes of green extraction, isolation, purification, and recovery to obtain greater quantities of healthy bioactive phytochemicals to discern the interaction within the food matrix. Nowadays, there is a clear need for potentially efficient natural products against COVID-19, which requires an in-depth study of the phytochemical potentials found in residues. Special attention has been given to flavonoids as natural substances that promote the prevention or recovery from SARS-CoV-2 infection due to the wide range of their biological effects, including the modulation of inflammatory processes and immune responses. At present, the fight against coronavirus has been conducted based on treatment with drugs traditionally used against other pathologies generated by viruses (HIV protease inhibitors, such as ritonavir and lopinavir, and anti-inflammatory agents, such as tocilizumab or dexamethasone); the bioactive compounds mentioned in this review, such as flavonoids, are a viable alternative since they lack systemic toxicity, generate a synergy with traditional drugs, and present a pleiotropic effect, since their functional groups can interact with different cellular targets and intercept multiple pathways. It is also important to point out that the use of these bioactive compounds as antivirals can have not only a curative but also a preventive effect since they can inhibit the proteases of the viruses, blocking their propagation.

With the current knowledge regarding the nutritional and phytochemical composition of winemaking residues, more and better research is needed to understand the composition and deliver accurate results for use and application of innovative products that contribute not only to the nutritional or medical field but are a viable alternative for saving time and reducing the environmental impact of wine production worldwide. Finally, it is important to point out that the characteristics of the by-products will depend on a variety of factors, mainly regarding grape production, agronomic management (irrigation, fertilization, etc.), the geographical place of origin, and the management inherent to wine production within the winery.

## Figures and Tables

**Table 3:** Studies on the health/biochemical properties of different bioactive extracts against some diseases and viruses.

Bioactive Ingredient Extract	Disease and Virus	Reference
Grape seed and grape marc meal extract	Gut morphology, apparent digestibility of nutrients, microbial composition in faeces, and the expression of pro-inflammatory genes in the intestine of pigs.	[48]
Extraction from wine production waste (seeds, skin, and pomace) from Pinot noir and Pinot meunier	Anti-influenza activity	[49]
Polyphenols extraction from Cabernet Sauvignon grape pomace	Effect of different classes of antibiotics against *Staphylococcus aureus* and *Escherichia coli*, especially against multi-drug resistant clinical isolates	[50]
Oligostilbenoids isolated from extracts of *Vitis vinifera* L. Pinot Noir grape canes	Antiproliferative activity on four different cell lines (MCR-5, AGS, SK-MES-1, and J82) determined by means of the MTT reduction assay.	[51]
Leaf extract *Vitis vinifera* var. Paulsen 1103	Antiviral activity against two human viruses: The Herpes simplex virus type 1 (HSV-1) and widespread severe acute respiratory syndrome coronavirus 2 (SARS-CoV-2).	[52]
Phenolic extract from grape stems (*Vitis vinifera* var. Red Globe)	Inhibit the growth of *Listeria monocytogenes*, *Staphylococcus aureus*, *Salmonella enterica* subsp. enterica serovar *Typhimurium*, and *Escherichia coli* O157	[53]
Hydroalcoholic extract from grape pomace var. Máximo IAC 138-22	Ovicidal and larvicidal activity against gastrointestinal nematodes of sheep.	[54]
Grape seed extract	Antiviral activities against hepatitis A virus (HAV) and human norovirus surrogates (feline calicivirus (FCV-F9) and murine norovirus (MNV-1)).	[55]
Grape seed-extracted proanthocyanidin	Inhibition of porcine reproductive and respiratory syndrome virus (PRRSV)	[56]

## Appendix A

**Table A1:** Main bioactive compounds found in pomace by-products.

Compounds of Interest	Grape Pomace (Skin and Seed)	Grape Skin	Grape Seed
Gallic acid	1090.1 μg g^−1^ of extract (RP-HPLC) [69]	122 μg g^−1^ of extract (HPLC–UV) [70]	9.8 mg kg^−1^ of fresh grape (HPLC-DAD-FLV) [71]
	397.67 μg mL^−1^ of extract (HPLC-DAD) [72]	8.76 mg kg^−1^ dw (UHPLC-DAD-MS/MS) [73]	30.3 mg kg^−1^ dw (RP-HPLC/UV) [74]
	252.8 μg g^−1^ of extract (HPLC-MWD) [8]	1.19 mg kg^−1^ of grape (HPLC-DAD) [75]	136.74 mg kg^−1^ dw (UHPLC-DAD-MS/MS) [73]
	95.36 mg kg^−1^ dw (HPLC-ESI/MS/MS) [76]		1.92 mg kg^−1^ of grape (HPLC-DAD) [75]
	260.92 mg L^−1^ of extract (HPLC-PDA-MS) [77]		
Syringic acid	1731.7 μg g^−1^ of extract (HPLC-MWD) [8]		
Caffeic acid	16.0 μg g^−1^ of extract (HPLC-MWD) [8]	0.54 mg kg^−1^ dw (UHPLC-DAD-MS/MS) [73]	1.06 mg kg^−1^ dw (UHPLC-DAD-MS/MS) [73]
	438.43 mg kg^−1^ dw (HPLC-ESI/MS/MS) [76]		
p-Coumaric acid	64.6 μg g^−1^ of extract (HPLC-MWD) [8]	1.96 mg kg^−1^ of grape (HPLC-DAD) [75]	
	214.55 mg kg^−1^ dw (HPLC-ESI/MS/MS) [76]		
Ferulic acid	24.1 μg g^−1^ of extract (HPLC-MWD) [8]	2.12 mg kg^−1^ dw (UHPLC-DAD-MS/MS) [73]	2.17 mg kg^−1^ dw (UHPLC-DAD-MS/MS) [73]
	1.33 mg kg^−1^ dw (HPLC-ESI/MS/MS) [76]		
Caftaric acid	1.80 mg kg^−1^ dw (HPLC-PDA-ESI-MS/MS) [78]		
Trans-resveratrol	36.0 μg g^−1^ of extract (HPLC-MWD) [8]	5.64 mg kg^−1^ dw (UHPLC-DAD-MS/MS) [73]	
	20.66 mg kg^−1^ dw (HPLC-ESI/MS/MS) [76]	1.43 mg kg^−1^ of grape (HPLC-DAD) [73]	
Cyanidin 3-*O*-glucoside	870 μg g^−1^ of extract (HPLC-MWD) [8]	528 mg kg^−1^ dw (UPLC-DAD-MS) [79]	
	6.99 mg kg^−1^ dw (HPLC-UV-DAD) [80]		
Myricetin	36.77 mg kg^−1^ dw (HPLC-ESI/MS/MS) [76]	1.8 μmol kg^−1^ of grape (HPLC-DAD/FLD) [81]	2.42 mg kg^−1^ dw (UHPLC-DAD-MS/MS) [73]
	452 ppm of dry extract (HPLC-DAD-ESI-MS/MS) [82]	2.1 mg kg^−1^ dw (UHPLC-DAD-MS/MS) [73]	
	2.45 mg kg^−1^ dw (HPLC-PDA-ESI-MS/MS) [78]		
Rutin	998.5 μg g^−1^ of extract (RP-HPLC) [69]	57.04 mg kg^−1^ dw (HPLC-DAD) [83]	9.05 mg kg^−1^ dw (HPLC-DAD) [83]
	112.96 μg mL^−1^ of extract (HPLC-DAD) [72]	223 μg g^−1^ of extract (HPLC–UV) [70]	30.7 mg kg^−1^ dw (RP-HPLC/UV) [74]
Delphinidin 3-*O*-acetylglucoside	1043 μg g^−1^ of extract (HPLC-MWD) [8]		
	9.79 mg L^−1^ of extract (HPLC-PDA-MS) [77]		
(+)-Catechin	5083 μg g^−1^ of extract (RP-HPLC) [69]	13.20 mg kg^−1^ dw (HPLC-DAD) [83]	117 mg kg^−1^ dw (HPLC-DAD) [83]
	89.73 mg kg^−1^ dw (HPLC-PDA-ESI-MS/MS) [78]	628 μg g^−1^ of extract (HPLC–UV) [70]	270 mg kg^−1^ of fresh grape (HPLC-DAD-FLV) [73]
	275.09 μg mL^−1^ of extract (HPLC-DAD) [72]	49.38 mg kg^−1^ of grape (HPLC–DAD–ESI-MS/MS) [84]	21.1 mg kg^−1^ dw (RP-HPLC/UV) [74]
	3387.5 μg g-1 of extract (HPLC-MWD) [8]	7.47 mg kg^−1^ dw (UHPLC-DAD-MS/MS) [73]	86.73 mg kg^−1^ of grape (HPLC–DAD–ESI-MS/MS) [84]
		11.45 mg kg^−1^ of grape (HPLC-DAD) [75]	270.26 mg kg^−1^ dw (UHPLC-DAD-MS/MS) [71]
		25 mg kg^−1^ of fresh grape (HPLC-DAD-FLV) [71]	106.5 mg kg^−1^ of grape (HPLC-DAD) [75]
(-)-Epicatechin	192.8 μg g^−1^ of extrac (RP-HPLC) [69]	323 μg g^−1^ of extract (HPLC–UV) [70]	210 mg kg^−1^ of fresh grape (HPLC-DAD-FLV) [71]
	1763.4 μg g^−1^ of extract (HPLC-MWD) [8]	13.55 mg kg^−1^ of grape (HPLC–DAD–ESI-MS/MS) [84]	38.1 mg kg^−1^ dw (RP-HPLC/UV) [74]
	112.72 mg kg^−1^ dw (HPLC-PDA-ESI-MS/MS (Lingua, 2016 #242)	3.56 mg kg^−1^ dw (UHPLC-DAD-MS/MS) [73]	6.81 mg kg^−1^ of grape (HPLC–DAD–ESI-MS/MS) [84]
		2.67 mg kg^−1^ of grape (HPLC-DAD) [75]	223.08 mg kg^−1^ dw (UHPLC-DAD-MS/MS) [73]
		13 mg kg^−1^ of fresh grape (HPLC-DAD-FLV) [71]	77.51 mg kg^−1^ of grape (HPLC-DAD) [75]
		47.50 mg kg^−1^ dw (HPLC-DAD) [83]	
Kaempferol	346.8 μg g^−1^ of extract (RP-HPLC) [69]	34.2 mg kg^−1^ dw (UPLC-DAD-MS) [79]	
	28.53 mg kg^−1^ dw (HPLC-ESI/MS/MS) [76]	0.41 μmol kg^−1^ of grape (HPLC-PDA-ESI-MS/MS) [78]	
	2.37 mg kg^−1^ dw (HPLC-UV-DAD) [80]	8.93 mg kg^−1^ dw (HPLC-DAD/FLD) [81]	
	34.23 mg kg^−1^ dw (HPLC-PDA-ESI-MS/MS) [78]	14.89 mg kg^−1^ dw (UHPLC-DAD-MS/MS) [73]	
		1.53 mg kg^−1^ dw (UPLC-DAD-MS) [79]	
Quercetin 3-glucuronide	130 mg kg^−1^ dw (HPLC-UV-DAD) [80]	22 mg kg^−1^ dw (UPLC-DAD-MS) [79]	
	81.42 mg kg^−1^ dw (HPLC-PDA-ESI-MS/MS) [78]	0.98 mg 100g^−1^ (HPLC-DAD) [75]	
	990 ppm of dry extract (HPLC-DAD-ESI-MS/MS) [82]		
Peonidin 3-*O*-glucoside	0,15 mg g^−1^ of extract (HPLC-UV-DAD) [85]	551 mg kg^−1^ dw (UPLC-DAD-MS) [79]	
	2460 μg g^−1^ of extract (HPLC-MWD) [8]		
	18.31 mg L^−1^ of extract (HPLC-PDA-MS) [77]		
	1591 ppm of dry extract (HPLC-DAD-ESI-MS/MS) [82]		
	18.70 mg kg^−1^ dw (HPLC-UV-DAD) [80]		
	0.97 mg kg^−1^ dw (HPLC-PDA-ESI-MS/MS) [78]		
Malvidin 3-*O*-glucoside	5,70 mg g^−1^ of extract (HPLC-UV-DAD) [85]	2489 mg kg^−1^ dw (UPLC-DAD-MS) [79]	
	26,658 μg g^−1^ of extract (HPLC-MWD) [8]		
	955.85 mg L^−1^ of extract (HPLC-PDA-MS) [77]		
	12182 ppm of dry extract (HPLC-DAD-ESI-MS/MS) [82]		
	64.6 mg kg^−1^ dw (HPLC-UV-DAD) [80]		
	142.22 mg kg^−1^ dw (HPLC-PDA-ESI-MS/MS) [78]		
Quercetin	650.2 μg g^−1^ of extract (RP-HPLC–DAD) [69]	316 μg g^−1^ of extract (HPLC–UV) [70]	1009.4 mg kg^−1^ dw (RP-HPLC/UV) [74]
	159.60 μg mL^−1^ of extract (HPLC-DAD) [72]	40.03 mg kg^−1^ dw (HPLC-DAD) [83]	11.72 mg kg^−1^ dw (UHPLC-DAD-MS/MS) [73]
	557.3 μg g^−1^ of extract (HPLC-MWD) [8]	0.53 μmol kg^−1^ of grape (HPLC-DAD/FLD) [81]	
	26.25 mg kg^−1^ dw (HPLC-ESI/MS/MS) [76]	121.94 mg kg^−1^ dw (UHPLC-DAD-MS/MS) [73]	
	382.93 mg L^−1^ of extract (HPLC-PDA-MS) [77]	1043 mg kg^−1^ dw (UPLC-DAD-MS) [79]	
	0.54 mg g^−1^ of extract (HPLC-UV-DAD) [85]	3.68 mg kg^−1^ dw (HPLC-DAD) [83]	
	392 ppm of dry extract (HPLC-DAD-ESI-MS/MS) [82]		
	15.30 mg kg^−1^ dw (HPLC-UV-DAD) [80]		
	251.06 mg kg^−1^ dw (HPLC-PDA-ESI-MS/MS) [78]		
Delphinidin 3-*O*-glucoside	0,16 mg g^−1^ of extract (HPLC-UV-DAD) [85]	870 mg kg^−1^ dw (UPLC-DAD-MS) [79]	
	4581 μg g^−1^ of extract (HPLC-MWD) [8]		
	4.47 mg L^−1^ of extract (HPLC-PDA-MS) [77]		
	775 ppm of dry extract (HPLC-DAD-ESI-MS/MS) [82]		
	3.73 mg kg^−1^ dw (HPLC-UV-DAD) [80]		
Petunidin 3-*O*-acetylglucoside	1424 μg g^−1^ of extract (HPLC-MWD) [8]		
	72.13 mg L^−1^ of extract (HPLC-PDA-MS) [77]		
	0.86 mg kg^−1^ dw (HPLC-PDA-ESI-MS/MS) [78]		
Malvidin 3-*O*-acetylglucoside	2,02 mg g^−1^ of extract (HPLC-UV-DAD) [85]	486 mg kg^−1^ dw (UPLC-DAD-MS) [79]	
	4021 μg g^−1^ of extract (HPLC-MWD) [8]		
	1718.92 mg L^−1^ of extract (HPLC-PDA-MS) [77]		
	937 ppm of dry extract (HPLC-DAD-ESI-MS/MS) [82]		
	0.96 mg kg^−1^ dw (HPLC-UV-DAD) [80]		
	195.01 mg kg^−1^ dw (HPLC-PDA-ESI-MS/MS) [78]		
Cyanidin 3-*O*-p-coumaroylglucoside	1886 μg g^−1^ of extract (HPLC-MWD) [8]	327 mg kg^−1^ dw (UPLC-DAD-MS) [79]	
	3.99 mg L^−1^ of extract (HPLC-PDA-MS) [77]		
Petunidin 3-*O*-p-coumaroylglucoside	2481 μg g^−1^ of extract (HPLC-MWD) [8]	339 mg kg^−1^ dw (UPLC-DAD-MS) [79]	
	29.95 mg L^−1^ of extract (HPLC-PDA-MS) [77]		
	765 ppm of dry extract (HPLC-DAD-ESI-MS/MS) [82]		
	72.95 mg kg^−1^ dw (HPLC-PDA-ESI-MS/MS) [78]		
Peonidin 3-*O*-acetylglucoside	1902 μg g^−1^ of extract (HPLC-MWD) [8]		
	32.64 mg L^−1^ of extract (HPLC-PDA-MS) [77]		
	1.83 mg kg^−1^ dw (HPLC-PDA-ESI-MS/MS) [78]		

## References

[1] Matos C., Pirra A. (2020). Water to wine in wineries in Portugal Douro Region: Comparative study between wineries with different sizes. Sci. Total Environ..

[2] OIV (2020). Wine Production First Estimates. 27 October 2020. https://www.greatwinecapitals.com/wp-content/uploads/2021/02/OIV-2020-World-Wine-Production-First-Estimates-presentation-pdf.pdf.

[3] Bharathiraja B., Iyyappan J., Jayamuthunagai J., Kumar R.P., Sirohi R., Gnansounou E., Pandey A. (2020). Critical review on bioconversion of winery wastes into value-added products. Ind. Crops Prod..

[4] Dwyer K., Hosseinian F., Rod M.R. (2014). The market potential of grape waste alternatives. J. Food Res..

[5] de Campos L.M., Leimann F.V., Pedrosa R.C., Ferreira S.R. (2008). Free radical scavenging of grape pomace extracts from Cabernet sauvingnon (Vitis vinifera). Bioresour. Technol..

[6] Beres C., Costa G.N., Cabezudo I., da Silva-James N.K., Teles A.S., Cruz A.P., Mellinger-Silva C., Tonon R.V., Cabral L.M., Freitas S.P. (2017). Towards integral utilization of grape pomace from winemaking process: A review. Waste Manag..

[7] Kekelidze I., Ebelashvili N., Japaridze M., Chankvetadze B., Chankvetadze L. (2018). Phenolic antioxidants in red dessert wine produced with innovative technology. Ann. Agrar. Sci..

[8] Antoniolli A., Fontana A.R., Piccoli P., Bottini R. (2015). Characterization of polyphenols and evaluation of antioxidant capacity in grape pomace of the cv. Malbec. Food Chem..

[9] Moro K.I.B., Bender A.B.B., da Silva L.P., Penna N.G. (2021). Green Extraction Methods and Microencapsulation Technologies of Phenolic Compounds From Grape Pomace: A Review. Food Bioprocess Technol..

[10] Barba F.J., Zhu Z., Koubaa M., Sant’Ana A.S., Orlien V. (2016). Green alternative methods for the extraction of antioxidant bioactive compounds from winery wastes and by-products: A review. Trends Food Sci. Technol..

[11] El Darra N., Grimi N., Maroun R.G., Louka N., Vorobiev E. (2013). Pulsed electric field, ultrasound, and thermal pretreatments for better phenolic extraction during red fermentation. Eur. Food Res. Technol..

[12] Stuper-Szablewska K., Perkowski J. (2019). Phenolic acids in cereal grain: Occurrence, biosynthesis, metabolism and role in living organisms. Crit. Rev. Food Sci. Nutr..

[13] Rashmi H.B., Negi P.S. (2020). Phenolic acids from vegetables: A review on processing stability and health benefits. Food Res. Int..

[14] Teixeira A., Baenas N., Dominguez-Perles R., Barros A., Rosa E., Moreno D.A., Garcia-Viguera C. (2014). Natural bioactive compounds from winery by-products as health promoters: A review. Int. J. Mol. Sci..

[15] Wu M., Luo Q., Nie R., Yang X., Tang Z., Chen H. (2021). Potential implications of polyphenols on aging considering oxidative stress, inflammation, autophagy, and gut microbiota. Crit. Rev. Food Sci. Nutr..

[16] Garrido J., Borges F. (2013). Wine and grape polyphenols—A chemical perspective. Food Res. Int..

[17] Bakowska-Barczak A.M., Kolodziejczyk P.P. (2011). Black currant polyphenols: Their storage stability and microencapsulation. Ind. Crops Prod..

[18] Sansone F., Mencherini T., Picerno P., d’Amore M., Aquino R.P., Lauro M.R. (2011). Maltodextrin/pectin microparticles by spray drying as carrier for nutraceutical extracts. J. Food Eng..

[19] Delgado-Torre M.P., Ferreiro-Vera C., Priego-Capote F., Pérez-Juan P.M., Luque de Castro M.a.D. (2012). Comparison of accelerated methods for the extraction of phenolic compounds from different vine-shoot cultivars. J. Agric. Food Chem..

[20] Tolun A., Altintas Z., Artik N. (2016). Microencapsulation of grape polyphenols using maltodextrin and gum arabic as two alternative coating materials: Development and characterization. J. Biotechnol..

[21] Tsali A., Goula A.M. (2018). Valorization of grape pomace: Encapsulation and storage stability of its phenolic extract. Powder Technol..

[22] Tolun A., Artik N., Altintas Z. (2020). Effect of different microencapsulating materials and relative humidities on storage stability of microencapsulated grape pomace extract. Food Chem..

[23] de Souza V.B., Thomazini M., de Carvalho Balieiro J.C., Fávaro-Trindade C.S. (2015). Effect of spray drying on the physicochemical properties and color stability of the powdered pigment obtained from vinification byproducts of the Bordo grape (Vitis labrusca). Food Bioprod. Processing.

[24] Gençdağ E., Özdemir E.E., Demirci K., Görgüç A., Yılmaz F.M. (2022). Copigmentation and stabilization of anthocyanins using organic molecules and encapsulation techniques. Curr. Plant Biol..

[25] Randazzo W., Fabra M.J., Falcó I., López-Rubio A., Sánchez G. (2018). Polymers and biopolymers with antiviral activity: Potential applications for improving food safety. Compr. Rev. Food Sci. Food Saf..

[26] Gómez-Mascaraque L.G., Sanchez G., López-Rubio A. (2016). Impact of molecular weight on the formation of electrosprayed chitosan microcapsules as delivery vehicles for bioactive compounds. Carbohydr. Polym..

[27] Rani J., Rautela A., Kumar S., Krishnaraj Rathinam N., Sani R.K. (2020). Chapter 4—Biovalorization of winery industry waste to produce value-added products. Biovalorisation of Wastes to Renewable Chemicals and Biofuels.

[28] Xia E.-Q., Deng G.-F., Guo Y.-J., Li H.-B. (2010). Biological activities of polyphenols from grapes. Int. J. Mol. Sci..

[29] Salehi B., Vlaisavljevic S., Adetunji C.O., Adetunji J.B., Kregiel D., Antolak H., Pawlikowska E., Uprety Y., Mileski K.S., Devkota H.P. (2019). Plants of the genus Vitis: Phenolic compounds, anticancer properties and clinical relevance. Trends Food Sci. Technol..

[30] Spinelli R., Nati C., Pari L., Mescalchin E., Magagnotti N. (2012). Production and quality of biomass fuels from mechanized collection and processing of vineyard pruning residues. Appl. Energy.

[31] Duranay N.D., Akkuş G. (2019). Solid fuel production with torrefaction from vineyard pruning waste. Biomass Convers. Biorefinery.

[32] Friedman M. (2014). Antibacterial, Antiviral, and Antifungal Properties of Wines and Winery Byproducts in Relation to Their Flavonoid Content. J. Agric. Food Chem..

[33] Royo C., Ferradás Y., Martínez-Zapater J.M., Motilva M.-J. (2021). Characterization of Tempranillo negro (VN21), a high phenolic content grapevine Tempranillo clone, through UHPLC-QqQ-MS/MS polyphenol profiling. Food Chem..

[34] Zhu L., Zhang Y., Lu J. (2012). Phenolic Contents and Compositions in Skins of Red Wine Grape Cultivars among Various Genetic Backgrounds and Originations. Int. J. Mol. Sci..

[35] Xu H., Liu X., Yan Q., Yuan F., Gao Y. (2015). A novel copigment of quercetagetin for stabilization of grape skin anthocyanins. Food chem..

[36] Salehi B., Mishra A.P., Nigam M., Sener B., Kilic M., Sharifi-Rad M., Fokou P.V.T., Martins N., Sharifi-Rad J. (2018). Resveratrol: A double-edged sword in health benefits. Biomedicines.

[37] Wilkowska A., Ambroziak W., Czyzowska A., Adamiec J. (2016). Effect of microencapsulation by spray-drying and freeze-drying technique on the antioxidant properties of blueberry (Vaccinium myrtillus) juice polyphenolic compounds. Pol. J. Food Nutr. Sci..

[38] Gessner D., Ringseis R., Eder K. (2017). Potential of plant polyphenols to combat oxidative stress and inflammatory processes in farm animals. J. Anim. Physiol. Anim. Nutr..

[39] Yadav K., Bajaj R.K., Mandal S., Mann B. (2020). Encapsulation of grape seed extract phenolics using whey protein concentrate, maltodextrin and gum arabica blends. J. Food Sci. Technol..

[40] Prado-Alvarez M., Lynch S., Kane A., Darmody G., Pardo B., Martínez P., Cotterill J., Wontner-Smith T., Culloty S. (2015). Oral immunostimulation of the oyster Ostrea edulis: Impacts on the parasite Bonamia ostreae. Fish Shellfish. Immunol..

[41] Krishnaiah D., Sarbatly R., Nithyanandam R. (2011). A review of the antioxidant potential of medicinal plant species. Food Bioprod. Processing.

[42] Sun Y., Liu Q., Xi B., Dai H. (2019). Study on the regulation of anthocyanin biosynthesis by exogenous abscisic acid in grapevine. Sci. Hortic..

[43] Yilmaz E.E., Özvural E.B., Vural H. (2011). Extraction and identification of proanthocyanidins from grape seed (Vitis Vinifera) using supercritical carbon dioxide. J. Supercrit. Fluids.

[44] Ky I., Lorrain B., Kolbas N., Crozier A., Teissedre P.-L. (2014). Wine by-products: Phenolic characterization and antioxidant activity evaluation of grapes and grape pomaces from six different French grape varieties. Molecules.

[45] Hassan F.A., Mahrose K.M., Basyony M.M. (2016). Effects of grape seed extract as a natural antioxidant on growth performance, carcass characteristics and antioxidant status of rabbits during heat stress. Arch. Anim. Nutr..

[46] Natarajan S.B., Hwang J.-W., Kim Y.-S., Kim E.-K., Park P.-J. (2017). Ocular promoting activity of grape polyphenols—A review. Environ. Toxicol. Pharmacol..

[47] Bekhit A.E.-D.A., Cheng V.J., McConnell M., Zhao J.H., Sedcole R., Harrison R. (2011). Antioxidant activities, sensory and anti-influenza activity of grape skin tea infusion. Food Chem..

[48] Fiesel A., Gessner D.K., Most E., Eder K. (2014). Effects of dietary polyphenol-rich plant products from grape or hop on pro-inflammatory gene expression in the intestine, nutrient digestibility and faecal microbiota of weaned pigs. BMC Vet. Res..

[49] Bekhit A.E.-D.A., Cheng V.J., Zhang H., Mros S., Mohamed Ahmed I.A., Al-Juhaimi F.Y., Bekhit A.A., McConnell M. (2019). Effect of extraction system and grape variety on anti-influenza compounds from wine production residue. Food Control.

[50] Sanhueza L., Melo R., Montero R., Maisey K., Mendoza L., Wilkens M. (2017). Synergistic interactions between phenolic compounds identified in grape pomace extract with antibiotics of different classes against Staphylococcus aureus and Escherichia coli. PLoS ONE.

[51] Sáez V., Pastene E., Vergara C., Mardones C., Hermosín-Gutiérrez I., Gómez-Alonso S., Gómez M.V., Theoduloz C., Riquelme S., von Baer D. (2018). Oligostilbenoids in Vitis vinifera L. Pinot Noir grape cane extract: Isolation, characterization, in vitro antioxidant capacity and anti-proliferative effect on cancer cells. Food Chem..

[52] Zannella C., Giugliano R., Chianese A., Buonocore C., Vitale G.A., Sanna G., Sarno F., Manzin A., Nebbioso A., Termolino P. (2021). Antiviral Activity of Vitis vinifera Leaf Extract against SARS-CoV-2 and HSV-1. Viruses.

[53] Vázquez-Armenta F., Silva-Espinoza B., Cruz-Valenzuela M., González-Aguilar G., Nazzaro F., Fratianni F., Ayala-Zavala J. (2017). Antibacterial and antioxidant properties of grape stem extract applied as disinfectant in fresh leafy vegetables. J. Food Sci. Technol..

[54] Silva Soares S.C., de Lima G.C., Carlos Laurentiz A., Féboli A., dos Anjos L.A., de Paula Carlis M.S., da Silva Filardi R., da Silva de Laurentiz R. (2018). In vitro anthelmintic activity of grape pomace extract against gastrointestinal nematodes of naturally infected sheep. Int. J. Vet. Sci. Med..

[55] Joshi S.S., Su X., D’Souza D.H. (2015). Antiviral effects of grape seed extract against feline calicivirus, murine norovirus, and hepatitis A virus in model food systems and under gastric conditions. Food Microbiol..

[56] Zhang M., Wu Q., Chen Y., Duan M., Tian G., Deng X., Sun Y., Zhou T., Zhang G., Chen W. (2018). Inhibition of proanthocyanidin A2 on porcine reproductive and respiratory syndrome virus replication in vitro. PLoS ONE.

[57] Braga A.R.C., Murador D.C., de Souza Mesquita L.M., de Rosso V.V. (2018). Bioavailability of anthocyanins: Gaps in knowledge, challenges and future research. J. Food Compos. Anal..

[58] Ghosh D., Konishi T. (2007). Anthocyanins and anthocyanin-rich extracts: Role in diabetes and eye function. Asia Pac. J. Clin. Nutr..

[59] Cheng Y.-C., Sheen J.-M., Hu W.L., Hung Y.-C. (2017). Polyphenols and oxidative stress in atherosclerosis-related ischemic heart disease and stroke. Oxidative Med. Cell. Longev..

[60] Pauletto M., Elgendy R., Ianni A., Marone E., Giantin M., Grotta L., Ramazzotti S., Bennato F., Dacasto M., Martino G. (2020). Nutrigenomic effects of long-term grape pomace supplementation in dairy cows. Animals.

[61] Ishida K., Kishi Y., Oishi K., Hirooka H., Kumagai H. (2015). Effects of feeding polyphenol-rich winery wastes on digestibility, nitrogen utilization, ruminal fermentation, antioxidant status and oxidative stress in wethers. Anim. Sci. J..

[62] Biscarini F., Palazzo F., Castellani F., Masetti G., Grotta L., Cichelli A., Martino G. (2018). Rumen microbiome in dairy calves fed copper and grape-pomace dietary supplementations: Composition and predicted functional profile. PLoS ONE.

[63] Kafantaris I., Kotsampasi B., Christodoulou V., Kokka E., Kouka P., Terzopoulou Z., Gerasopoulos K., Stagos D., Mitsagga C., Giavasis I. (2017). Grape pomace improves antioxidant capacity and faecal microflora of lambs. J. Anim. Physiol. Anim. Nutr..

[64] Loaiza-Cano V., Monsalve-Escudero L.M., Martinez-Gutierrez M., Sousa D.P.d. (2021). Antiviral Role of Phenolic Compounds against Dengue Virus: A Review. Biomolecules.

[65] Lin S.-C., Ho C.-T., Chuo W.-H., Li S., Wang T.T., Lin C.-C. (2017). Effective inhibition of MERS-CoV infection by resveratrol. BMC Infect. Dis..

[66] Konowalchuk J., Speirs J.I. (1976). Virus inactivation by grapes and wines. Appl. Environ. Microbiol..

[67] Messina G., Polito R., Monda V., Cipolloni L., Di Nunno N., Di Mizio G., Murabito P., Carotenuto M., Messina A., Pisanelli D. (2020). Functional role of dietary intervention to improve the outcome of COVID-19: A hypothesis of work. Int. J. Mol. Sci..

[68] Roshanravan N., Seif F., Ostadrahimi A., Pouraghaei M., Ghaffari S. (2020). Targeting Cytokine Storm to Manage Patients with COVID-19: A Mini-Review. Arch. Med. Res..

[69] Sagdic O., Ozturk I., Ozkan G., Yetim H., Ekici L., Yilmaz M.T. (2011). RP-HPLC–DAD analysis of phenolic compounds in pomace extracts from five grape cultivars: Evaluation of their antioxidant, antiradical and antifungal activities in orange and apple juices. Food Chem..

[70] Farhadi K., Esmaeilzadeh F., Hatami M., Forough M., Molaie R. (2016). Determination of phenolic compounds content and antioxidant activity in skin, pulp, seed, cane and leaf of five native grape cultivars in West Azerbaijan province, Iran. Food Chem..

[71] Montealegre R.R., Peces R.R., Vozmediano J.C., Gascueña J.M., Romero E.G. (2006). Phenolic compounds in skins and seeds of ten grape Vitis vinifera varieties grown in a warm climate. J. Food Compos. Anal..

[72] Ribeiro L., Ribani R., Francisco T., Soares A., Pontarolo R., Haminiuk C. (2015). Profile of bioactive compounds from grape pomace (Vitis vinifera and Vitis labrusca) by spectrophotometric, chromatographic and spectral analyses. J. Chromatogr. B.

[73] Pantelić M.M., Zagorac D.Č.D., Davidović S.M., Todić S.R., Bešlić Z.S., Gašić U.M., Tešić Ž.L., Natić M.M. (2016). Identification and quantification of phenolic compounds in berry skin, pulp, and seeds in 13 grapevine varieties grown in Serbia. Food Chem..

[74] Tounsi M.S., Ouerghemmi I., Wannes W.A., Ksouri R., Zemni H., Marzouk B., Kchouk M.E. (2009). Valorization of three varieties of grape. Ind. Crops Prod..

[75] Di Lecce G., Arranz S., Jáuregui O., Tresserra-Rimbau A., Quifer-Rada P., Lamuela-Raventós R.M. (2014). Phenolic profiling of the skin, pulp and seeds of Albariño grapes using hybrid quadrupole time-of-flight and triple-quadrupole mass spectrometry. Food Chem..

[76] Ramirez-Lopez L., McGlynn W., Goad C., DeWitt C.M. (2014). Simultaneous determination of phenolic compounds in Cynthiana grape (Vitis aestivalis) by high performance liquid chromatography–electrospray ionisation–mass spectrometry. Food Chem..

[77] Trikas E.D., Melidou M., Papi R.M., Zachariadis G.A., Kyriakidis D.A. (2016). Extraction, separation and identification of anthocyanins from red wine by-product and their biological activities. J. Funct. Foods.

[78] Lingua M.S., Fabani M.P., Wunderlin D.A., Baroni M.V. (2016). In vivo antioxidant activity of grape, pomace and wine from three red varieties grown in Argentina: Its relationship to phenolic profile. J. Funct. Foods.

[79] Harsha P.S., Gardana C., Simonetti P., Spigno G., Lavelli V. (2013). Characterization of phenolics, in vitro reducing capacity and anti-glycation activity of red grape skins recovered from winemaking by-products. Bioresour. Technol..

[80] Amico V., Napoli E., Renda A., Ruberto G., Spatafora C., Tringali C. (2004). Constituents of grape pomace from the Sicilian cultivarNerello Mascalese’. Food Chem..

[81] Gómez-Alonso S., García-Romero E., Hermosín-Gutiérrez I. (2007). HPLC analysis of diverse grape and wine phenolics using direct injection and multidetection by DAD and fluorescence. J. Food Compos. Anal..

[82] Drosou C., Kyriakopoulou K., Bimpilas A., Tsimogiannis D., Krokida M. (2015). A comparative study on different extraction techniques to recover red grape pomace polyphenols from vinification byproducts. Ind. Crops Prod..

[83] Rockenbach I.I., Gonzaga L.V., Rizelio V.M., Gonçalves A.E.d.S.S., Genovese M.I., Fett R. (2011). Phenolic compounds and antioxidant activity of seed and skin extracts of red grape (Vitis vinifera and Vitis labrusca) pomace from Brazilian winemaking. Food Res. Int..

[84] Rebello L.P.G., Lago-Vanzela E.S., Barcia M.T., Ramos A.M., Stringheta P.C., Da-Silva R., Castillo-Muñoz N., Gómez-Alonso S., Hermosín-Gutiérrez I. (2013). Phenolic composition of the berry parts of hybrid grape cultivar BRS Violeta (BRS Rubea× IAC 1398-21) using HPLC–DAD–ESI-MS/MS. Food Res. Int..

[85] Ruberto G., Renda A., Daquino C., Amico V., Spatafora C., Tringali C., De Tommasi N. (2007). Polyphenol constituents and antioxidant activity of grape pomace extracts from five Sicilian red grape cultivars. Food Chem..

